# *Nursing Reports* Annual Report Card 2025

**DOI:** 10.3390/nursrep16010001

**Published:** 2025-12-19

**Authors:** Richard Gray

**Affiliations:** School of Nursing and Midwifery, La Trobe University, Melbourne, VIC 3086, Australia; r.gray@latrobe.edu.au

For those of you who adhere to the Gregorian calendar, December marks the end of the year and a time for celebration and some well-earned rest. Overall, 2025 has been a busy and important year for those of us involved with *Nursing Reports*, and so in this editorial I wanted to celebrate some of this year’s achievements and consider some of the emergent issues that both authors and editors need to address going forward (including, of course, artificial intelligence).

I wanted to begin by thanking our colleagues who have supported the journal this year, particularly all authors and reviewers, the Managing Editor, the Editorial Assistants, the Editorial Board Members and our section Editors-in-Chief; without your support, there would be no journal.

I have been the Editor-in-Chief of *Nursing Reports* (published by MDPI) for just over five years. In my first year we published just 23 papers, and I think it is probably safe to say that *Nursing Reports* was not uppermost in the minds of authors when they were considering where to publish their next paper. In 2025, we published well over 400 papers (for the statistically minded, this represents an increase of approximately 1700% in five years), making *Nursing Reports* one of the biggest journals—in terms of numbers of papers published—in the field of nursing. This impressive achievement positions the journal as one of the most influential in the discipline. Perhaps more importantly, the number of people that read the papers we publish has also grown exponentially; in 2025, the papers published in *Nursing Reports* had an estimated 236,811 readers from 212 countries. For me, this is a key metric of success; after all, why publish a paper if no one will read it?

The issue of access to scientific knowledge is an important one—*Nursing Reports*, like all MDPI journals, is Open Access. Most clinical research is supported by public funds, yet there is a palpable sense of frustration [1] that the people who pay for research cannot view the outcomes because they are hidden behind a publisher paywall. By publishing in an Open Access journal, anyone—be they health professionals, researchers or members of the public—can freely read every paper.

The issue of access to academic papers (almost) seamlessly links to the next topic I would like to discuss: public involvement. The involvement of people with lived experience is increasingly recognized as a foundational aspect of clinical research, making the work more inclusive, transparent and impactful. In an editorial published in the journal earlier this year [2], we highlighted how nursing seemingly lags behind other clinical disciplines with regard to how we involve people with lived experience in our research. As an editorial team, we want to advocate for greater public involvement in nursing science. To this end, we are one of the few nursing journals that require authors to report how people with lived experience were involved in the work submitted for publication. By doing so, we aim to encourage our colleagues to consider how this involvement will enhance their research going forward.

No editorial about academic scholarship in 2025 would be complete without mention of the influence of artificial intelligence (AI), both in terms of the writing and review of papers. AI can be a force for good—for example, it can assist authors without English as a first language in editing their work to ensure clarity, precision and ease of reading. That said, over the course of this year I have noticed a marked increase in the number of papers submitted to the journal that were clearly partly or wholly written by AI. In turn, this can lead to important errors in reporting, unintentional plagiarism and the inclusion of fake (hallucinated) citations, raising further concerns. Any authors doing so should be aware that, as an editorial team, we are vigilant toward the use of AI and require the inclusion of a statement during the submission process regarding AI usage in the drafting and editing of manuscripts. Where AI has been used but not declared (bearing in mind that we are effective at spotting this), the manuscript will be rejected.

The use of AI in the review of manuscripts has also emerged as a point of concern over the course of 2025. I regularly receive reviews that have obviously been written using AI, whether in full or in part. Peer review is a confidential process; reviewers are not permitted to disclose the content of the manuscript they are reviewing to anyone else. If a reviewer uploads a manuscript to an AI platform, this represents a major breach of author confidentiality as they are, essentially, publishing work that the AI may then use for other purposes. In my experience, AI reviews of manuscripts also tend to be of extremely poor quality, providing bland and uninformative summaries of the work without the level of critical detail that is expected in peer review. As with drafting manuscripts, AI may be helpful for editing feedback to authors (for example, checking tone, clarity of language and precision of comments), which is an appropriate use of the technology. As an editorial team, we have a zero-tolerance policy regarding AI-generated peer review; where it occurs, the review will be disregarded and the reviewer removed from our database. For more information regarding our research and publication ethics guidelines, please visit https://www.mdpi.com/ethics#_bookmark4 (accessed on 14 December 2025).

I also wanted to comment on the number of reviews of the literature, particularly scoping reviews, submitted to *Nursing Reports* this year. Senior editorial colleagues from other journals have expressed concern about the emergence of almost production-line levels of seemingly ‘pointless’ or ‘redundant’ literature reviews [3], an observation that we would tend to agree with. We have also noted an apparent dramatic increase in the number of scoping reviews published, which seem to have become the dominant form of review in nursing science. Recently, the scoping reviews [4] published in *Nursing Reports* this year were audited; overall, they tended to be somewhat niche, included few studies, rarely engaged with stakeholders (an important part of scoping review methodology) and often made recommendations for policy and practice beyond their limited remit. Whilst we will continue to welcome scoping reviews for submission, we do intend, as an editorial policy, to take a much more critical stance in terms of determining their contribution to knowledge and adherence to the relevant guidelines.

Finally, I will outline some notable achievements for *Nursing Reports* in 2025. First, four new sections have been introduced, each led by a Section Editor-in-Chief. In doing so, we aimed to collate and draw attention to important emerging areas in nursing science under the editorial leadership of experts in the field. These four sections are listed below:

- Mental Health Nursing (Section Editor-in-Chief: Prof. Dr. Daniel Bressington).
- Nursing Care for Older People (Section Editor-in-Chief: Prof. Dr. Alessandro Stievano).
- Nursing Education and Leadership (Section Editor-in-Chief: Dr. Piyanee Klainin Yobas).
- Artificial Intelligence and Digital Innovations in Nursing Care (Section Editor-in-Chief: Dr. Niall S. Higgins).

In 2025, we recruited 15 scholars to our Early Career Editorial Board. This is an important new initiative that the *Nursing Reports* editorial team is extremely proud of. In establishing an Early Career Editorial Board, we aim to provide insights, training and support to a new generation of nursing journal editors. We welcome our new colleagues and look forward to working with them though 2026 and beyond.

This year, *Nursing Reports* has supported several key nursing conferences across the globe, including The National Nursing Forum, The 30th International Mental Health Nurse Research Conference, American Public Health Association 2025, GSA 2025 Annual Scientific Meeting, The 34th International Congress—Science of Paediatric and Neonatal Intensive Care, The 27th Annual Trauma Care Conference and the International Congress of Nutrition. We also hosted three reviewer clubs (in April, July and October) with the aim of providing a forum to discuss emerging issues and topics in peer review; this year, topics included questionable research practices, the importance of pre-registration of research, and how to craft a positive peer review.

Each year, *Nursing Reports* provides awards for the two ‘best papers’ and outstanding reviewers. It was my privilege to chair the judging panel. The 2025 awards are shown below:

- Best research paper: Petros Galanis, for their paper comparing burnout in nurses with other healthcare workers following the pandemic [5].
- Best review paper: Lisa Reid, for their narrative review ‘Challenging the Myth of the Digital Native’ [6].
- Outstanding reviewers: Dragana Milutinović, University of Novi Sad, Serbia, and Wendy Lee Sarver, Cleveland State University, USA.

A third award, the Editor of Distinction Award, will be introduced in 2026, which will be given to an Editorial Board Member who has made exceptional contributions to the journal.

Over the past five years, I believe that *Nursing Reports* has established itself as a leading Open Access nursing journal. As Editor-in-Chief, I am indebted to all reviewers and authors and my editorial colleagues for their contributions to the journal. As an editorial team, we are committed to providing authors with a professional, positive, and efficient service. We look forward to working with you further in 2026.
